# Impact of a Structured CAARMS Workshop on Trainee Confidence and Knowledge in Assessing At-Risk Mental States

**DOI:** 10.1192/bjo.2026.11323

**Published:** 2026-06-30

**Authors:** Kaynat Riaz, Imtiaz Ahmad Dogar, Samreen Afzal, Iqra Yousaf

**Affiliations:** Allied II Hospital, Faisalabad Medical University, Faisalabad, Pakistan

## Abstract

**Aims:**

To evaluate the educational impact of a structured CAARMS workshop on trainee confidence and knowledge related to the assessment of at-risk mental states.

**Methods:**

A pre-post educational evaluation was conducted among participants attending a CAARMS workshop. Anonymous surveys were completed immediately before and after the session. Self-reported confidence and clinical skills were assessed using a workshop-specific questionnaire aligned with core CAARMS competencies, including symptom elicitation, differentiation of attenuated psychotic symptoms, threshold determination, risk assessment, formulation, and documentation. Knowledge was assessed using a curriculum-aligned multiple-choice questionnaire based on key CAARMS principles and common assessment pitfalls. Anonymous participant codes enabled paired analysis. Post-workshop feedback assessed perceived relevance and applicability to clinical practice.

**Results:**

Paired pre-post analysis demonstrated improvements across all assessed domains. The proportion of participants rating themselves as highly confident (≥7/10) increased from 28% pre-workshop to 76% post-workshop. The largest gains were observed in differentiating attenuated psychotic symptoms from anxiety, obsessive-compulsive phenomena, and culturally normative beliefs (22% to 74%), applying CAARMS threshold criteria (18% to 71%), and conducting structured risk assessments (31% to 82%). Knowledge performance improved, with 83% of participants achieving high post-training scores compared with 41% pre-training. Workshop acceptability was high, with 92% reporting the content as relevant to their clinical work and 94% indicating they would recommend the workshop to other trainees.

**Conclusion:**

A structured, case-based CAARMS workshop was associated with substantial improvements in trainee confidence and knowledge related to at-risk mental state assessment. This simple pre-post evaluation approach provides a practical method for assessingeducational impact and may support earlier and more accurate identification of individuals at risk of psychosis across psychiatric training settings.

